# Inter- and intra-annual differences in foraging ecology of the chick-rearing Brünnich’s guillemots (*Uria lomvia*) breeding in the High Arctic

**DOI:** 10.1098/rsos.250932

**Published:** 2025-09-10

**Authors:** Karolina Cieślińska, Lech Marek Iliszko, Michał Goc, Lech Stempniewicz, Dariusz Jakubas

**Affiliations:** ^1^ Department of Vertebrate Ecology and Zoology, University of Gdańsk, Gdańsk, Poland

**Keywords:** central-place foraging, colony, GPS tracking, niche partitioning, remote sensing, temporal prey depletion halo

## Abstract

The foraging ecology of seabirds depends on both external and internal factors. Seabirds can modify their feeding strategy depending on current food availability to maintain optimal energy levels provisioned to the offspring. Here, we investigated inter- and intra-annual variability of the foraging ecology of the Brünnich’s guillemot (*Uria lomvia*) breeding in a High Arctic colony on Spitsbergen (Svalbard) combining GPS tracking and remote sensing. Despite different environmental conditions in the studied years, covered distances and duration of foraging trips were similar. The studied individuals generally foraged in cold waters at shelf and shelf break zones located up to 100 km from the colony (median 51 km). They foraged at colder waters with lower primary productivity in colder 2015 compared with warmer 2016 but still used areas of similar depth. They explored a narrower foraging habitat niche (described by sea surface temperature, chlorophyll *a* concentration, sea depth and seabed slope in foraging locations) in warmer 2016, suggesting a lower variety of microhabitats where the preferred prey was available. With progress of the chick-rearing period, they foraged further from the colony, suggesting temporal prey depletion halo effect. Our findings provide valuable insight into spatio-temporal variability of seabird foraging ecology in the rapidly changing High Arctic.

## 1. Introduction

Energy and time allocation differs considerably in seabirds parents depending on both external (environmental conditions, distribution and availability of prey, weather, stage of breeding season) and internal factors (sex, age, body and health condition) [1–6]. Marine birds can be capable of buffering unfavourable conditions to maintain optimal energy supply to their growing offspring by exploring various foraging microhabitats and modifying foraging strategy, e.g. by changing trip length and duration or diving tactics (e.g. [7–10]). They can buffer suboptimal condition until some threshold beyond which they may suffer from lower breeding success or adult survival (e.g. [11,12]). These may affect population dynamics (e.g. [13]) and finally lead to local extirpation (e.g. [14]).

Inter- and intra-specific competition is inevitable for seabirds when food resources around the colony and time for breeding are limited [2,10,15]. Conditions on the feeding grounds experienced by birds during foraging can be defined as foraging habitat niche [16]. According to the theory, two similar species or two sexes of a single species cannot share the same niche due to strong competition [17]. Partitioning of resources in space (e.g. foraging in different habitats, areas or depths of the water column), time (e.g. foraging at different times of the day) and exploiting different type of prey, relieves negative outcomes of this relation, enabling a relatively stable coexistence in shared ecosystem by two similar species or sexes [17,18]. However, foraging effort is expected to primarily target resources and strategies providing maximal reward in a given environment [19]. Consequently, within the same habitat, species or individuals are on average more likely to forage for the same abundance/quality of resources, resulting in the resource niche overlap [20]. It is especially possible in a highly productive habitats such as shelf break zone, oceanographic fronts, eddies or tidal glacier bays where high concentrations of individuals of the same or/and different species sharing the same foraging guild are observed (e.g. [21–27]). The breadth of the foraging habitat niche varies greatly in response to the dynamic changes occurring in the marine environment. Those variations are based on temporal and spatial food availability, and are frequently experienced by seabirds in polar regions.

High Arctic seabirds often have low tolerance for environmental conditions (e.g. [4]), especially those that alter the availability of preferred prey [2]. These include sea temperature, salinity and chlorophyll *a* concentration (CHLA) (an indicator of primary water productivity and prey aggregations [28,29]). For this reason, Arctic seabirds constitute a good model group for studying the response of marine vertebrates to dynamic changes in the marine environment. These changes are driven by macroscale fluctuations of oceanographic conditions (e.g. North Atlantic Oscillation [30]) as well as mesoscale processes including sea ice dynamics, sea current distribution, upwelling strength and spread [31–35] and recently by rapid climatic changes in the Arctic [36–41]. An example of a High Arctic seabird sensitive to environmental changes is the Brünnich’s guillemot (or thick-billed murre, *Uria lomvia*) (hereafter guillemot). Guillemots have the highest wing loading of any flying bird and therefore their flight is very costly [42–45]. They are pursuit divers feeding at great depths even over 100 m (e.g. [46,47]). This species is considered a feeding generalist [48], frequently foraging on cold-water or sea-ice-associated prey (e.g. [49–51]). Adult guillemots feed mainly on fish (polar cod *Boreogadus saida* and in lesser degree capelin *Mallotus villosus*), amphipods (*Themisto* sp.), but also on euphausiids (*Thysanoessa* sp.) or polychaetes [52,53]. As central-place foragers, guillemots are associated with the breeding colony during the chick-rearing period, which limits their foraging area [48,54,55]. Consequently, guillemots trade off energy spent on travel and foraging when energy costs and gains differ between available prey [56]. Adult guillemots, while being strictly dependent on foraging conditions at sea in proximity to a colony, modify characteristics of foraging flights to forage at the most optimal areas to efficiently feed themselves and their offspring (e.g. [57]). Variation in foraging ecology has been observed at spatial, i.e. geographical scale and may be expressed by different foraging effort due to different food availability around particular colonies [58]. Variation has been also reported at temporal, within-breeding season scale, i.e. between incubation and chick-rearing periods [59,60], and with the progress of chick-rearing period due to growth of energetical needs during offspring development [60,61] and/or depletion of food resources in the close proximity of the colony. The last phenomenon is characterized by increasing the foraging trips distance with the progress of the chick-rearing period, a phenomenon called ‘temporal prey depletion halo’ [47,59,60].

Focusing on the foraging ecology of the Brünnich’s guillemot, we investigated the intra- and inter-annual variability of foraging trip characteristics in response to environmental changes. To this end, we analysed the intra- and inter-annual variability of foraging trips characteristics (i.e. total duration, maximal range and total distance covered) and distance between the colony and foraging areas. We also investigated foraging habitat niche breadth (defined by remote-sensed environmental conditions important for guillemot’s prey: sea surface temperature (hereafter SST)), CHLA (primary production marker), sea depth (hereafter DEPTH), seabed slope (hereafter SLOPE)] of the chick-rearing GPS-tracked Brünnich’s guillemots breeding in the High Arctic colony on southwestern Spitsbergen in two consecutive breeding seasons differing in environmental conditions.

We tested three hypotheses:

(1) Given the heterogeneity of environmental conditions in the study area (presence of cold and warm water masses [62] and guillemot preferences for cold-water prey [52,63,64], we expected birds to forage mainly in cold-water areas.
(2) Given a year-to-year variability of environmental conditions in the study area [65–67], we expect inter-annual differences in foraging trip characteristics and habitat foraging niches realized by guillemots. In a year with suboptimal environmental conditions for a preferred cold-water prey, we expected broader foraging niches of guillemots reflecting seeking for alternative foraging microhabitats offering attractive prey.
(3) Given a possibility of depletion of food resources available close to the colony with a progress of the breeding season, we expected parental birds to perform longer foraging trips (in duration and distance) to maintain similar energy level provided to their offspring (temporal prey depletion halo effect) [47,59,60].

## 2. Material and methods

### 2.1. Study area

**Figure 1.**
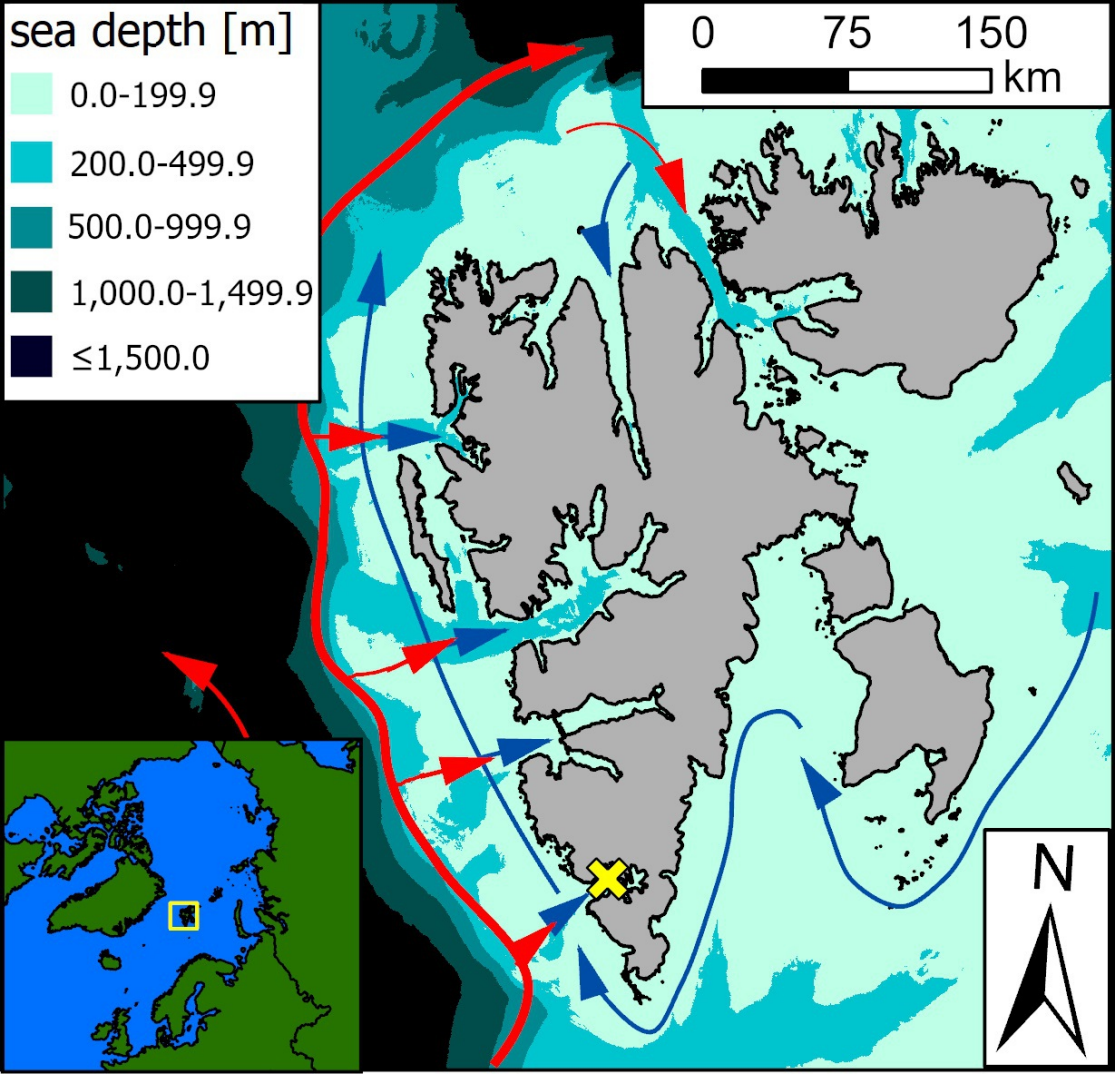
Location of the study area on Spitsbergen (main map; colony at Gnålodden cliffs in Hornsund marked with a yellow cross) and in the Arctic (inset map). Arrows indicate sea currents carrying Arctic-origin (blue) and Atlantic-origin (red) water masses. Sea currents distribution according to [68]. Source of bathymetry background map: IBCAO v. 3.0 500 m RR grid [69]. Map generated in ArcGIS Pro 3.2.2 [70], Svalbard land area (a major map) by the Norwegian Polar Institute (S100 Kartdata) [71].

We performed the study in Brünnich’s guillemots breeding colony at Gnålodden cliffs in Hornsund (SW Spitsbergen, Svalbard) estimated at 5500−10 000 breeding pairs (Norwegian Polar Institute, unpublished database). Hornsund is a High Arctic, medium-sized fjord, located in the southernmost part of the Spitsbergen western coast [27,66] (figure 1). It is 35 km long, 2−12 km wide and 90 m deep on average [66]. Its entrance is shallow and lacks a sill, making this fjord vulnerable towards inflow of outside waters [72,73]. The Hornsund area is influenced by two water currents, i.e. the coastal Sørkapp Current carrying cold Arctic water and West Spitsbergen Current transporting warm Atlantic water [66,72] (figure 1). These two distinct water masses are usually separated by a hydrological front (Arctic or Polar Front) situated on the shelf break zone. The shelf zone outside is influenced by cold-water masses [65,67] and constitutes an important foraging area for seabirds (e.g. [26]). The frontal zone can be rich in zooplankton (e.g. [73]) providing good foraging conditions for seabirds [74]. Hornsund fjord provides rich variety of feeding microhabitats due to its glaciation [27] combined with a diverse and iced coastline [25,26,66]. Environmental conditions in the Hornsund area vary between and within years (e.g. [27,65–67,75]).

### 2.2. Fieldwork and GPS tracking

We conducted fieldwork during the chick-rearing period (July) in 2015 and 2016. We caught parent birds on nest shelves using a fishing rod with a loop made of nylon line (1 mm thick) at the end. We put the loop around the neck of the selected bird and after tightening it, we pulled it off the shelf. Captured birds were weighed with a spring balance (accurate to 10 g) and measured. Then we deployed GPS loggers (five types (weight): URIA100 (8.5 g), URIA100 Solar (8.5 g), URIA240 (12 g), URIA240 Solar (12 g); ALLE100 (6 g), all produced by ECOTONE, Sopot, Poland). The loggers’ weight (including attachment = 1 g) constituted 0.6–1.5% of instrumented individuals’ body mass (given body mass ranged in captured individuals from 805 to 1065 g) and was concordant with generally accepted recommendation that the weight of the device should not exceed *ca* 3% of a bird’s body mass [76]. GPS loggers were deployed on back of individuals’ back feathers using stripes of Tesa tape (code 4965, Tesa Tape Inc., Charlotte, NC, USA). We released the instrumented birds after 10−15 min of handling. Birds were marked with a colour marker to enable checking if they returned to the nest.

We set GPS loggers to record locations every 15 min and to activate after the first contact with salt water (i.e. diving). Otherwise, recording started after 48 h. Data from GPS loggers were automatically downloaded to the base station located in the colony. Whenever birds with deployed loggers were within the base station range (approx. 200 m), GPS signal searching by the logger was automatically blocked to save battery power. Reactivation of GPS loggers always occurred whenever birds left the range of the base station. In total, we deployed loggers on 22 individuals, 7 in 2015 and 15 in 2016. Data from all GPS loggers deployed on individuals were downloaded to the base station in the colony indicating regular returns to the colony (it was confirmed by observation of the marked individuals in the nest ledges). The duration of GPS data recording lasted from 11 to 23 July in 2015 and from 8 to 30 July in 2016. Individual guillemots were tracked between 1 to 11 days (mean ± s.d.: 6.9 ± 4.00 days) in 2015 and from 2 to 22 days (mean ± s.d.: 11.3 ± 6.39 days) in 2016. We excluded from the analyses one individual in 2015 because it lost the logger shortly after deployment (see electronic supplementary material, table S1 for full report on individual GPS tracking). We performed all statistical and geospatial analyses in R software v. 4.2.2-3 [77]. We created maps in ArcGIS Pro 3.2.2 software [70].

### 2.3. GPS data analyses

To identify and calculate the basic foraging metrics of trips, we used the *track2KBA* package (v. 1.1.2 [78]). Foraging trip duration is highly variable in guillemots (e.g. [79–81]). In this study, as a complete trip we considered the one starting and ending within a 1 km radius buffer around the colony and lasting at least 3 h. We did not consider cases of incomplete trips, i.e. situations in which the GPS signal was temporarily lost during returning or after leaving the colony. Such cases prevented the algorithm from identifying a complete flight (*n* = 2 cases in 2015). Chosen duration threshold is equal to the minimal duration reported for the guillemot (e.g. [81]). To calculate distances between foraging locations of GPS-tracked individuals and the colony we used the *geosphere* package (v. 1.5-18 [82]). With the use of the *repAssess* function from this package, we assessed the representativeness of the study samples collected in both years, which was similar for both years (0.31 for 2015 and 0.38 for 2016). Despite the difference in sample size between the two years, the similarity in their representativeness suggests that they are unlikely to have affected any observed inter-annual differences.

Based on the GPS-loggers data, we calculated the following metrics for each foraging trip: (i) the maximum range of flight (km), i.e. distance covered from the colony to the most distal point of the trip; (ii) the total covered distance (km), interpreted as a cumulation of covered distances between all GPS locations along the track; (iii) total trip duration (h), recognized as time spent outside of the colony measured between departure and return of each individual to the colony during the single foraging trip; (iv) stationary locations (hereafter called ‘foraging locations’)—the locations with momentary speed less than 10 m s^−1^; such stationary locations suggest foraging behaviour as low transit speed is commonly considered as an indicator of foraging behaviour of marine predators (e.g. [83,84]); (v) diving events based on recorded number of contacts with the salt water (diving detection given position of the logger on the bird’s back). In analyses regarding foraging locations and frequency of diving events, we only included trips containing complete information for all environmental variables under consideration. Given the duration of the chick-rearing period and the chick growth stages (e.g. [45]), we included a day of the year (DOY) (sequential day number starting with day 1 on 1 January) as a proxy for the chick-rearing progress/chicks’ age.

We performed a mixed permutational analysis of variance (MPANOVA) to compare the foraging metrics between the years. Trip and bird identities were set as a random factor. We set number of permutations to 5000 and used *Rd_kheradPajouh_renaud* resampling method described by Kherad-Pajouh and Renaud [85,86]. We performed MPANOVA in the *permuco* package (v. 1.1.3 [87]). To compare differences in diving intensity between the studied years, we used generalized linear mixed model (GLMM, family Poisson) with a number of dives per trip in each tracked individual as a response variable, year as a factorial, DOY as a continuous explanatory variable, year:DOY interaction and bird and trip identity as random factors. GLMM was performed using the *glmer* function in the *lme4* package (v. 1.1−35.3 [88]). We used Akaike’s information criterion for small sample sizes (AICc) to select the best GLMM model [89,90]) with combinations of predictors included in the global model using the *dredge* function) in the *MuMIn* package (v. 1.48.11 [91]). We compared the relative performance of the models based on ∆AICc, i.e. the difference between the AIC value of the best model and the AIC value for each of the other models [89]. We selected as the best model the one with the lowest ∆AIC.

### 2.4. Remote-sensed environmental conditions in foraging areas

To characterize environmental conditions at the foraging locations, we used remote-sensed data (SST and CHLA) from the moderate-resolution imaging spectroradiometer (MODIS) onboard the Aqua satellite [92]. We used monthly mosaics of level 3 data from this mission [93]. Both CHLA (mg m^−3^) and SST (°C) products consist of 4 km grid with 0.042° vertical and horizontal resolution. We chose SST since it is known to affect the large-scale distribution of the species [93] as guillemots prefer cold-water prey [52,64,94]. High concentrations of CHLA indicate areas of high productivity serving as hot spot zones for foraging predators [95]. We also included bathymetric features: DEPTH and SLOPE because of their role in driving stratification regimes and prey aggregations [96]. We extracted bathymetry features for the foraging locations from the International Bathymetric Chart of the Arctic Ocean v. 5 (IBCAO) of resolution of 100 m [97]. We calculated SLOPE based on the mentioned IBCAO v. 5 model with the spatial frame of eight neighbouring raster cells using the *terra* package (v. 1.7−18 [98]).

Using MPANOVA, we compared environmental conditions on the foraging grounds (SST, CHLA, DEPTH and SLOPE) between the studied years. We also used MPANOVA to compare the distances between the foraging locations and the colony in the 2 years of the study. We set bird/trip identity as a random factor and used *Rd_kheradPajouh_renaud* resampling method described by Kherad-Pajouh and Renaud [85,86] with default number of 5000 permutations. We performed MPANOVA in the *permuco* package (v. 1.1.3 [87]).

To investigate preferences of guillemots towards particular foraging habitats, we compared environmental conditions (SST, CHLA, DEPTH and SLOPE) between foraging locations and random points (we sampled 10 times more random points than observed GPS locations) within the annual home range (95% aKDE; see §2.5) (electronic supplementary material, figure S1), using the *amt* package [99]. As we expected nonlinear relationship, we performed generalized additive models (GAM, family binomial) with location type (foraging locations coded as 1 and random locations coded as 0), smoothed environmental variable and tensor product interaction between latitude and longitude of foraging and random locations (to control for spatial autocorrelation) as explanatory variables. We computed GAMs in the *mgcv* package (v. 1.9-1 [100]) and visualized the *visreg* package (v. 2.7.0 [101]).

### 2.5. Core and home ranges

To pinpoint the most important foraging areas, we calculated core and home ranges using autocorrelated kernel density estimation (aKDE) [102,103], based on stationary, i.e. foraging, locations of GPS-tracked individuals. aKDE is currently considered as the most efficient and the only proper technique dealing with autocorrelated tracking data [103]. Unlike other KDEs, aKDE is not based on indirect approach, which led to biases during area estimation [103]. Conventionally used methods do not consider the temporal autocorrelation inherited in modern tracking data, nor the uncertainty of individual’s home-range estimate [104]. aKDE derives several confidence intervals (estimated value, high-, low-weighted estimates), which appropriately reflect the limited precision of modelled estimates [103]. We calculated mean annual home (95% aKDE) and core (50% aKDE) ranges in the *ctmm* package (v. 1.2.0 [105]).

We compared between the studied years home and core ranges using MPANOVA based on ranges of particular individuals. We performed MPANOVA in the *permuco* package (v. 1.1.3 [87]) with the number of permutations set to 5000 and using the Rd_kheradPajouh_renaud resampling method [85,86]. To evaluate the size of mutually exploited foraging areas (95% aKDE), we calculated Bhattacharyya’s affinity (BA) overlap index between years [106]. BA is an efficient overlap estimator, fulfilling most of the expected estimator criteria [107] and being widely used in ecological studies (e.g. [108]). This estimator quantifies the relative similarity (overlap) between two statistical samples, where 1 indicates identical distributions and 0 stands for no similarity between investigated samples. We calculated BA in the *ctmm* package (v. 1.2.0 [105]).

### 2.6. Foraging habitat niche breadth

According to theory, the fundamental niche of a species is determined by its physiological range of tolerance to environmental factors in the absence of biotic interactions [109]. However, spatio-temporal variability in various factors, including environmental ones, may reduce the fundamental niche to a smaller realized niche [110]. In this study, we compared the realized foraging habitat niche of guillemots in the studied years differing in environmental conditions (SST, CHLA, DEPTH and SLOPE) at the foraging locations of the GPS-tracked individuals. We calculated niche breadth in the Bayesian inference framework using the *nicheROVER* package [111,112]. We created niches as regions of 95% probability and at 1000 runs (for higher results accuracy). Finally, we calculated the breadth of foraging niches, which enabled us to compare their mutual overlap [111]. We compared the computed foraging niches using two niche region sizes (95 and 99%) [112]. We compared niche breadth between the studied years using Wilcoxon test in the *rstatix* package (v. 0.7.2 [113]) based on the niches of individual birds. To visualize the calculated niches, we used two-dimensional scatterplots with ellipses depicting 10 random projections of the foraging niches in two-dimensional perspectives defined by two variables.

### 2.7. Factors affecting foraging locations characteristics

To investigate the temporal prey depletion halo effect, we performed linear mixed-effects models (LMMs) with distance between the colony and foraging locations as a response variable, year (factorial variable), day of the year (DOY; a proxy of advancement of the chick-rearing period; continuous variable) and year:DOY interaction as an explanatory variable. We set bird and trip identity as random effects. Similarly, we also used LMMs to investigate how environmental condition in foraging areas (SST, CHLA, DEPTH and SLOPE; response variables) were associated with year (factorial variable), DOY (continuous variable) and year:DOY interaction. Bird and trip identity were used as random effects. We calculated LMMs using *lmer* function in the *lme4* package (v. 1.1-35.3 [88]) on log-transformed values of environmental variables. We visualized LMMs in the *ggplot2* package (v. 3.5.2 [114]). We used AICc to select the best LMMs [89,90] with combinations of predictors included in the global model using the *dredge* function in the *MuMIn* package (v. 1.48.11 [91]). We compared the relative performance of the models based on ∆AICc, i.e. the difference between the AIC value of the best model and the AIC value for each of the other models [89]. We selected as the best model the one with the lowest ∆AIC.

## 3. Results

### 3.1. Foraging trips characteristics

We found no significant inter-annual differences in characteristics of foraging trips and distances (MPANOVA; all resampled *p* > 0.05; 5000 permutations for all cases). We found that the GPS-tracked guillemots during the chick-rearing period performed foraging trips with maximal range ranging from 2.4 to 100.2 km (median 41.8 km, 25–75% percentiles: 11.2−66.4 km), with the total distance covered by birds ranging from 7.5 to 357.8 km (median 92.3 km, 25–75% percentiles: 29.3−147.7 km) and the trip duration ranging from 3.0 to 53.7 h (median 9.8 h, 25–75% percentiles: 6.6−15.0 h). Distances between foraging locations and the colony in both years ranged between 1.0 to 100.2 km (median 51.4 km, 25−75% percentiles: 29.9−65.9 km) (table 1).

**Table 1.** Characteristics of foraging trips and locations of the GPS-tracked chick-rearing Brünnich’s guillemots breeding in Hornsund (SW Spitsbergen) in 2015 and 2016 (mean ± s.d.). MPANOVA, mixed permutational analysis of variance.

**year**	**foraging trip:**			distances (km)	number of:		
	duration (h)	total distance covered (km	maximal range (km	between foraging locations and the colony	GPS-tracked individuals	foraging locations	trips (max per ind.)
2015	11.6 ± 10.22	98.8 ± 81.75	39.1 ± 30.21	41.1 ± 59.68	6	1543	37 (10)
2016	12.2 ± 7.75	94.9 ± 63.30	41.3 ± 27.54	45.7 ± 39.76	15	4241	120 (17)
difference (MPANOVA)	*F* = 0.16, *p* = 0.69	*F* = 0.10, *p* = 0.75	*F* = 0.18, *p* = 0.67	*F* = 1.25, *p* = 0.27	—	—	—

### 3.2. Home and core ranges of foraging areas

**Figure 2.**
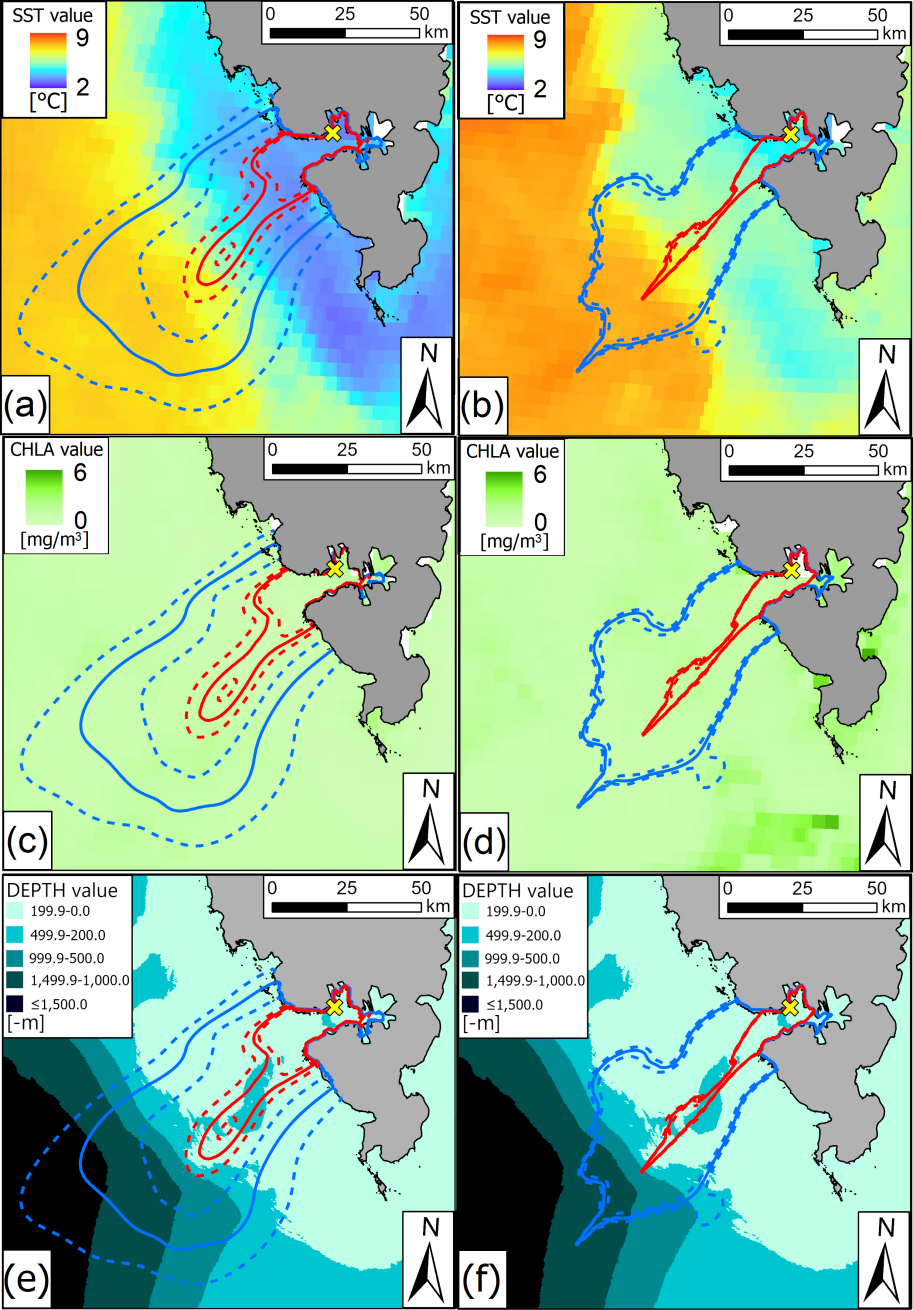
Autocorrelated KDE for foraging locations of the GPS-tracked chick-rearing Brünnich’s guillemots breeding in Hornsund (SW Spitsbergen) in 2015 (a,c,e) and 2016 (b,d,f). Red lines indicate 50% aKDE (core area), while blue lines stand for 95% aKDE (home range). Continuous lines depict estimated aKDE values, while dashed lines stand for high and low confidence intervals. aKDEs were visualized on raster with gradient values of environmental variables: SST in July (a,b), CHLA in July (c,d) and DEPTH (e,f). Yellow cross indicates the Gnålloden colony. White spots in SST and CHLA rasters indicate lack of data. Map generated in ArcGIS Pro 3.2.2 [70], Svalbard land area (a major map) by the Norwegian Polar Institute (S100 Kartdata) [71]).

GPS-tracked individuals in warmer 2016 utilized significantly smaller core range areas (mean ± s.d.: 312.6 ± 200.4 km^2^), compared with colder 2015 (mean ± s.d.: 721.9 ± 522.0 km^2^) (MPANOVA, *F* = 7.087, *p* = 0.01), and tended to have smaller home-range areas (1630.2 ± 933.4 km^2^) than in colder 2015 (2783.2 ± 1954.6 km^2^) (MPANOVA, *F* = 3.458, *p* = 0.07). Inter-annual BA overlap for estimated aKDE areas was 0.85 (CI: 0.83−0.86) (figure 2).

### 3.3. Environmental conditions in foraging locations

During both study years, guillemots generally foraged in water masses of low temperature (SST mean ± s.d.: 5.7 ± 1.37°C), intermediate CHLA (mean ± s.d.: 1.2 ± 0.31 mg m^−3^), in mesopelagic water zone (DEPTH mean ± s.d.: 241.9 ± 228.17 m) with intermediate values of SLOPE (mean ± s.d.: 2.0 ± 2.29°).

**Figure 3.**
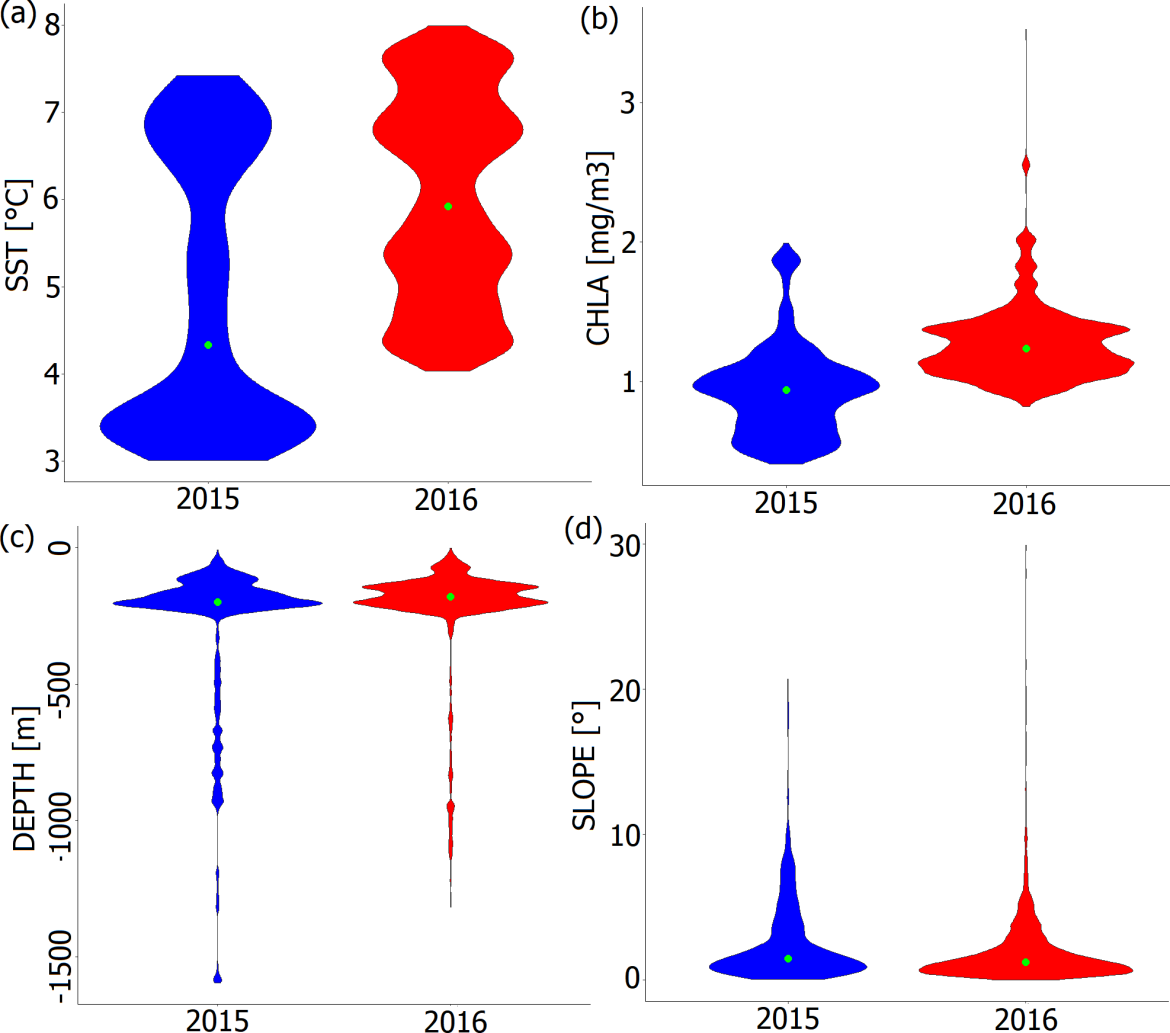
Environmental variables (a) SST; (b) CHLA; (c) DEPTH; (d) SLOPE in foraging locations of the chick-rearing GPS-tracked Brünnich’s guillemots breeding in Hornsund (SW Spitsbergen) in 2015 (*n =* 1543 foraging locations, blue) and 2016 (*n =* 4241 foraging locations, red). Violin plots represent the data density with the green dot in the middle standing for median.

We found that environmental variables in foraging locations differed significantly between the studied years with lower SST, CHLA and higher SLOPE in 2015 (hereafter the colder year) compared with 2016 (hereafter the warmer year) (figure 3, table 2). In contrast, DEPTH at foraging locations was similar in both studied years (figure 3, table 2).

**Table 2.** Environmental conditions in foraging locations of the GPS-tracked chick-rearing Brünnich’s guillemots breeding in Hornsund (SW Spitsbergen) in 2015 (*n =* 1543 foraging locations) and 2016 (*n =* 4241): SST, sea surface temperature; CHLA, chlorophyll *a* concentration; DEPTH, sea depth; SLOPE, seabed slope, IQR interquartile range.

		SST (°C)	CHLA (mg m^−3^)	DEPTH (m)	SLOPE (°)
MPANOVA	*F* *p*	28.84; <0.0001	88.86; <0.0001	3.497; 0.06	9.245; 0.002
2015	min–max	3.0−7.4	0.4−2.0	1595.2−7.1	0.04−20.7
	mean ± s.d.	4.9 ± 1.57	0.9 ± 0.32	292.0 ± 284.81	2.6 ± 2.81
	median; IQR	4.3; 3.34	0.9; 0.39	198.5; 72.51	1.5; 2.73
2016	min–max	4.0−8.0	0.8−3.5	1317.2−1.7	0.01−29.9
	mean ± s.d.	7.0 ± 1.16;	1.27 ± 0.26	223.7 ± 200.64	1.8 ± 2.0
	median; IQR	5.9; 1.97	1.2; 0.30	180.0; 73.04	1.2; 1.62

We fitted a GLMM to investigate how diving intensity changes between years of study, DOY and interaction between year and DOY. The best model included all variables considered, which were significant. We found that studied birds dived more frequently in colder 2015 (mean ± s.d.: 33.89 ± 23.83; *n* = 37 trips of six individuals), than in warmer 2016 (mean ± s.d.: 22.46 ± 18.31; *n* = 112 trips of 15 individuals) (GLMM; *z* = 2.948; *p* = 0.003). As the chick-rearing period progressed, birds dived more frequently (GLMM, *z* = 2.957; *p* = 0.003). The interaction effect was also significant, showing a more rapid increase in diving intensity of birds during colder 2015 compared with warmer 2016 (GLMM, *z* = −3.071, *p* = 0.002).

### 3.4. Environmental conditions preferences

**Figure 4.**
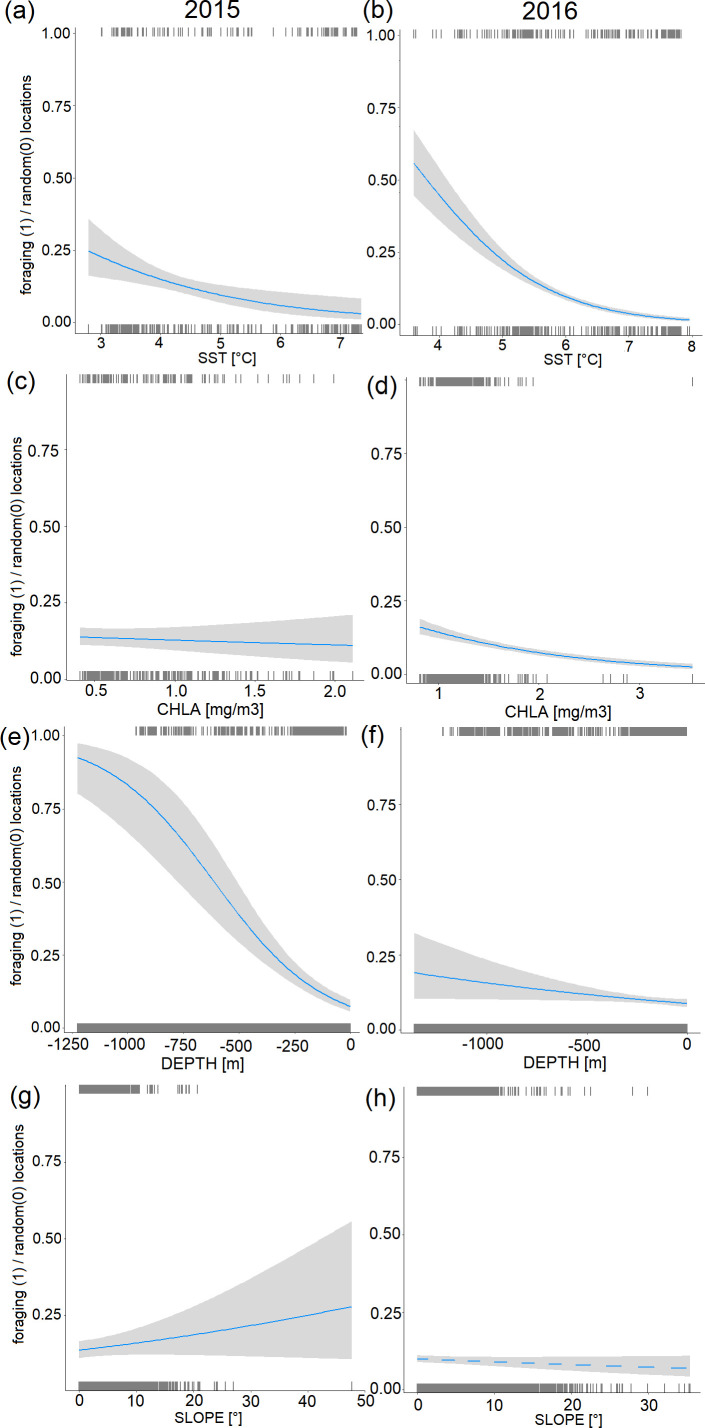
Relationships between location type (random coded as 0 and foraging as 1) of Brünnich’s guillemots breeding in Hornsund (SW Spitsbergen) in 2015 (1469 foraging versus 14 690 random locations, a,c,e,g) and 2016 (4481 foraging versus 44 810 random locations, b,d,f,h) and (a,b) SST, (c,d) CHLA, (e,f) DEPTH and (g,h) SLOPE. Solid lines indicate significant GAM fit, while dashed line indicates insignificant GAM fit. Rug plots indicate negative (bottom) and positive (top) residuals.

In all GAM models comparing environmental conditions at foraging locations of the GPS-tracked individuals, smoothed environmental variables and tensor product interactions between latitude and longitude of foraging and random locations were significant (all *p* ≤ 0.02) except for SLOPE in 2016 (*p* = 0.08) (table 3). We found that during both years of study, tracked guillemots preferred foraging in waters with lower SST, compared with random locations (GAM, *p* < 0.0001) (figure 4a,b, table 3). In 2015, guillemots preferred foraging in waters with higher CHLA (figure 4c), while in warmer 2016 they selected less productive water masses (GAM, *p* < 0.0001), compared with random locations (figure 4d, table 3). Also, in both years, guillemots used areas of shallower waters (GAM, *p* < 0.0001) (figure 4e,f, table 3). In colder 2015, they also foraged in areas characterized by steeper seabed (GAM, *p* = 0.02) (figure 4g). However, no significant effect of SLOPE was noted for warmer 2016 (figure 4h, table 3).

**Table 3.** Results of GAM for environmental conditions in foraging locations of the GPS-tracked chick-rearing Brünnich’s guillemots and randomly sampled locations within annual home ranges (aKDE 95%) in 2015 (1469 foraging locations versus 14 690 random points) and 2016 (4481 versus 44 810). Smoothed (s) environmental variables: SST, sea surface temperature; CHLA, chlorophyll *a* concentration; DEPTH, sea depth; SLOPE, seabed slope; XY, tensor product interaction between latitude and longitude of foraging and random locations; edf, effective degrees of freedom; ref.df, reference degrees of freedom; dev expl, deviance explained. Significant terms are bolded.

variable	year	variable	edf	ref.df	*F*	*p*	adj *R*^2^	dev expl
SST (°C)	2015	s(SST)	8.665	8.967	96.88	**<0.0001**	0.148	19.6%
		*t*(XY)	20.147	21.068	829.30	**<0.0001**		
	2016	s(SST)	8.811	8.991	209.4	**<0.0001**	0.0848	12.1%
		*t*(XY)	23.976	23.999	996.6	**<0.0001**		
CHLA (mg m^−3^)	2015	s(CHLA)	8.647	8.959	60.28	**<0.0001**	0.15	19.6%
		*t*(XY)	20.353	21.209	1015.51	**<0.0001**		
	2016	s(CHLA)	7.295	8.246	49.75	**<0.0001**	0.0657	10.7%
		*t*(XY)	23.983	24.0	1539.38	**<0.0001**		
DEPTH (m)	2015	s(DEPTH)	8.188	8.539	190.1	**<0.0001**	0.171	21.1%
		*t*(XY)	20.293	21.151	1088.2	**<0.0001**		
	2016	s(DEPTH)	8.101	8.717	240.2	**<0.0001**	0.0868	12.4%
		*t*(XY)	23.960	23.998	1934.3	**<0.0001**		
SLOPE (°)	2015	s(SLOPE)	7.258	8.29	24.3	**0.002**	0.139	18.9%
		*t*(XY)	20.467	21.28	1153.6	**<0.0001**		
	2016	s(SLOPE)	7.825	8.642	16.36	0.1	0.0789	11.5%
		*t*(XY)	23.970	23.999	2410.39	**<0.0001**		

### 3.5. Foraging habitat niche breadth

**Figure 5.**
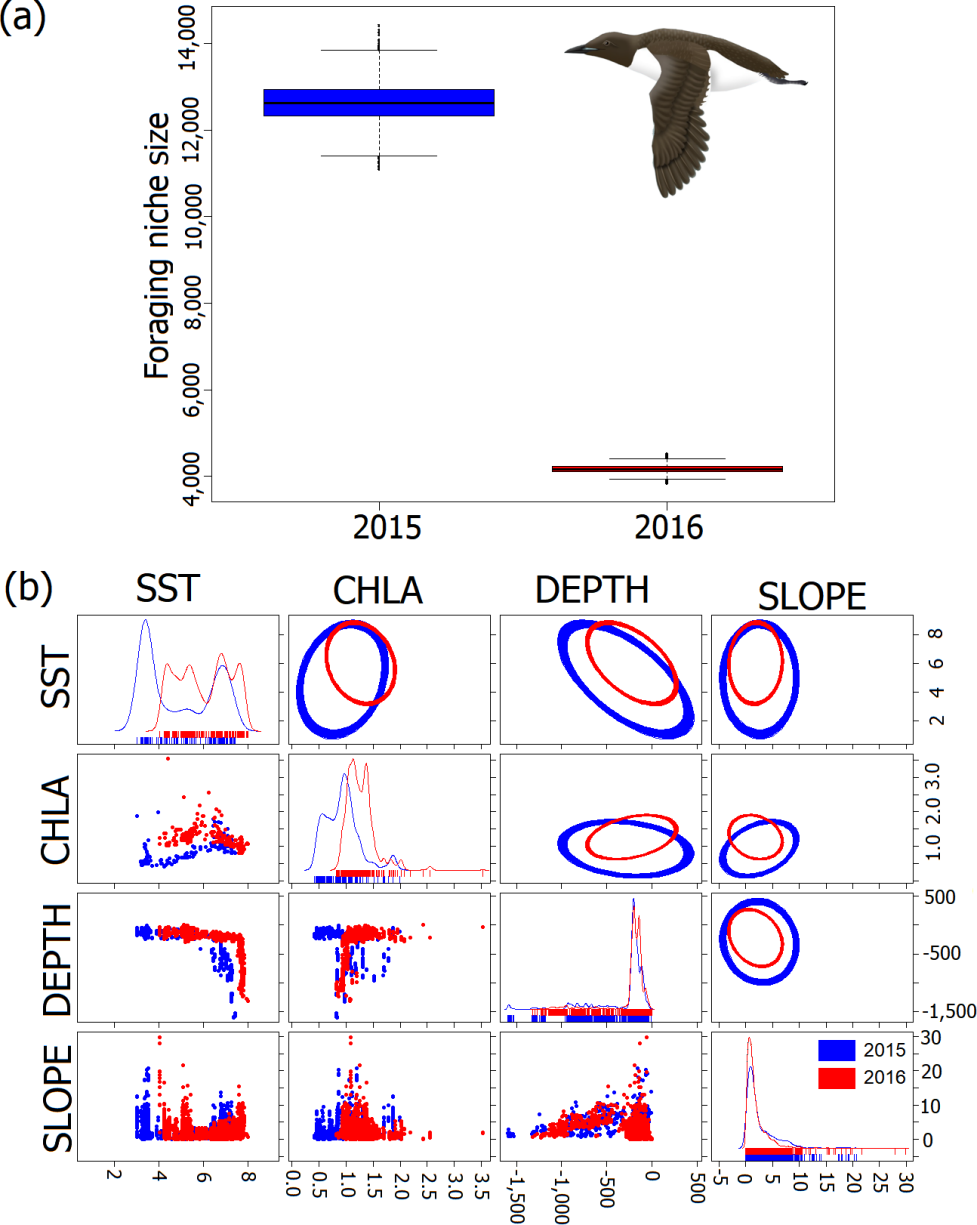
Foraging habitat niche breadths (a) of the GPS-tracked chick-rearing Brünnich’s guillemots breeding in Hornsund (SW Spitsbergen) in colder 2015 (blue) and warmer 2016 (red), and (b) their components (SST , CHLA , DEPTH and SLOPE in foraging locations, *n =* 1543 foraging locations in 2015 and *n =* 4241 in 2016). The distribution of environmental variables is displayed in the form of one-dimensional density plots. Two-dimensional scatterplots with ellipses represent trophic niches in two-dimensional perspectives of two variables. Boxplots show median (band inside the box), the first (25%) and third (75%) quartile (box), the lowest and the highest values within 1.5 interquartile range (whiskers) and outliers (dots). The guillemot’s graphic by K.C.

Realized foraging habitat niches of the GPS-tracked individuals in colder 2015 were significantly broader compared with warmer 2016 (Wilcoxon test; V = 2 001 000; *p* < 0.0001) (figure 5a; table 4). Distribution of SST in foraging locations was characterized by two peaks at approximately 3 and 7°C in colder 2015. In warmer 2016, four peaks were present at approximately 4, 5, 7 and 8°C SST (figure 5b). In colder 2015, we observed peaks in CHLA distribution with lower values (observed peaks at 0.5 and 1 mg m^−3^), compared with warmer 2016 (peaks for 1 and 1.5 mg m^−3^) (figure 5b). DEPTH distribution in foraging locations was generally similar in both years with most of the values corresponding to shelf zone depths (approx. 0–250 m). In 2015 more values corresponding to intermediate (approx. 500 m) and greater depths (less than approx. 500 m) were present, while in warmer 2016, prevalence for the shallow depths were present (less than approx. 200 m) (figure 5b). SLOPE distribution was also similar in both years with common peak around 1° and 2.5°. Additionally, in colder 2015, more values of steeper slope were observed (5−10°) (figure 5b).

**Table 4.** Foraging niche breadth of the GPS-tracked chick-rearing Brünnich’s guillemots breeding in Hornsund (SW Spitsbergen) in 2015 and 2016 and inter-annual overlap (95 and 99% niche region sizes).

year		**2015**	**2016**
mean		126 283.08	41 734.04
s.e.		4487.65	912.12
95% overlap	2015	—	49.11
	2016	87.86	—
99% overlap	2015	—	67.49
	2016	96.08	—

### 3.6. Intra- and inter-seasonal changes in foraging ecology

**Figure 6.**
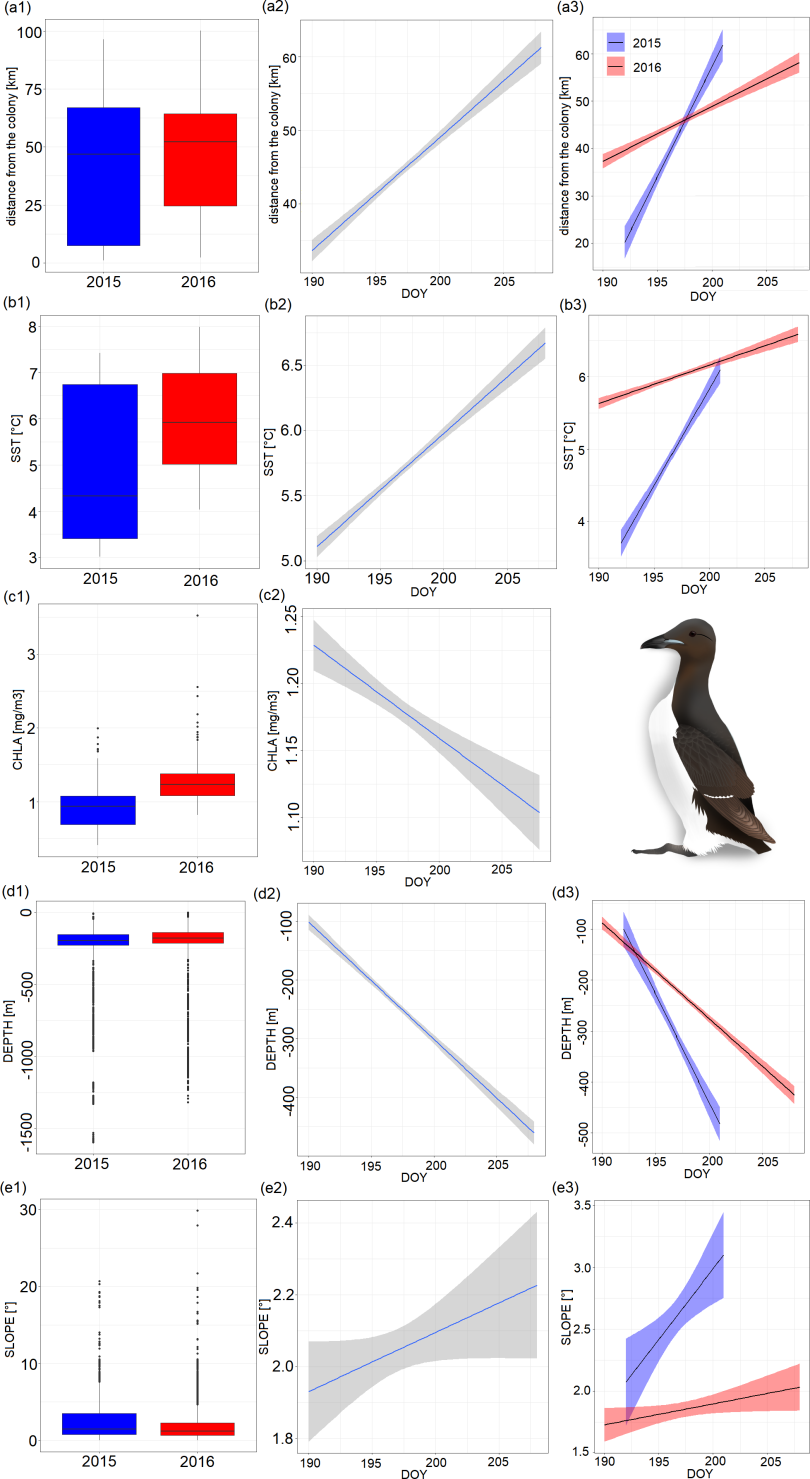
Factors affecting trip/environmental variables of the GPS-tracked chick-rearing Brünnich’s guillemots breeding in Hornsund (SW Spitsbergen) [(a) distance between foraging locations and the colony, (b) SST, (c) CHLA, (d) DEPTH, (e) SLOPE]. (a–e1) Boxplots for the studied years showing median (band inside the box), the first (25%) and third (75%) quartile (box), the lowest and the highest values within 1.5 interquartile range (whiskers) and outliers (dots). (a–e2) Relationship between the studied variables (a–e) and day of the year (DOY). Solid lines indicate significant LMM trends, ribbons around confidence intervals. (a,b3, d,e3) Interaction between year and DOY. Solid lines indicate significant LMM trends, ribbons around confidence intervals. Guillemot graphic by K.C.

We fitted an LMM to investigate how the distance between the colony and foraging locations changes between years of study, DOY and interaction between year and DOY. The best model included all variables considered. LMM revealed that the effects of all variables considered were significant (table 5). In warmer 2016, guillemots foraged further from the colony than in colder 2015 (figure 6a1). Distance between the foraging locations and the colony significantly increased with the progress of the chick-rearing period (figure 6a2, table 5). The interaction effect was also significant, showing more rapid increase of the distance between the foraging locations and the colony with the progress of the chick-rearing period in colder 2015 compared with warmer 2016 (figure 6a3, table 5).

**Table 5.** Results of LMMs for factors affecting trip/environmental variables of the GPS-tracked chick-rearing Brünnich’s guillemots breeding in Hornsund (SW Spitsbergen): year (2015, 2016), day of the year (DOY) and their interaction. Response variables: COLDIST, distance between foraging locations and the colony; SST, sea surface temperature; CHLA, chlorophyll *a* concentration; DEPTH, sea depth; SLOPE, seabed slope. Significant terms are bolded. Abbreviations: CI, confidence interval; EST, estimated value; s.e. standard error; adj. *R,* adjusted *R*^2^. Only the highest-ranked LMMs are presented.

response variables	predictors	EST	s.e.	*t*	CI	*p*	adj *R*
COLDIST (km)	year	14.93	1.383	10.80	12.22, 17.64	**<0.001**	0.78
	DOY	0.14	0.009	16.02	0.12, 0.16	**<0.001**	
	interaction	−0.0746	0.007	−10.58	−0.09, −0.06	**<0.001**	
SST (°C)	year	6.22	0.336	18.50	5.56, 6.88	**<0.001**	0.79
	DOY	0.04	0.002	19.60	0.04, 0.05	**<0.001**	
	interaction	−0.03	0.001713	−18.11	−0.03, −0.03	**<0.001**	
CHLA (mg m^−3^)	year	0.167	0.00422	39.402	0.16, 0.17	**<0.001**	0.60
	DOY	−0.005653	0.001204	−4.693	−0.008, −0.0033	**<0.001**	
DEPTH (m)	year	17.43	0.955	18.26	15.56, 19.30	**<0.001**	0.67
	DOY	0.11	0.006	18.64	0.10, 0.12	**<0.001**	
	interaction	−0.09	0.004866	−18.34	−0.10, −0.08	**<0.001**	
SLOPE (°)	year	9.57	1.92	4.986	5.81, 13.33	**<0.001**	0.32
	DOY	0.05	0.001	4.54	0.03, 0.07	**<0.001**	
	interaction	−0.0497	0.009784	−5.086	−0.07, −0.03	**<0.001**	

We also used LMMs to predict how environmental properties on the foraging sites change between years and DOY. For most of the environmental variables considered, models with year, DOY and their interactions were the ones with the best fit. Only in the case of CHLA the highest ranked model included only effects of year and DOY. Variables from all considered models had significant effect on environmental variables (figure 6, table 5). Guillemots also exploited warmer waters in warmer 2016 (figure 6b1). The studied individuals utilized warmer water masses with the progress of the chick-rearing period (figure 6b2, table 5). The interaction effect was also significant showing more rapid increase in SST with the progress of the chick-rearing period in colder 2015 compared with warmer 2016 (figure 6b3, table 5). The tracked individuals exploited waters of higher primary productivity in warmer 2016 than in colder 2015 (figure 6c1). As the chick-rearing period progressed, they also utilized locations with lower CHLA (figure 6c2, table 5). During the colder 2015, guillemots foraged more often in areas of greater depths than in 2016 (figure 6d1). The studied birds exploited areas of greater depths as chick-rearing period advanced (figure 6d2, table 5). Interaction effect was also significant, indicating more rapid decrease in DEPTH with the advancement of the chick-rearing period in colder 2015 compared with warmer 2016 (figure 6d3, table 5). Tracked guillemots used areas of steeper seabed while foraging in colder 2015 (figure 6e1). The tracked birds utilized areas of steeper seabed as chick-rearing period progressed (figure 6e2, table 5). Significant interaction effect exhibiting more rapid increase in SLOPE with the advancement of the chick-rearing period in colder 2015 compared with warmer 2016 (figure 6e3, table 5).

## 4. Discussion

Knowledge of the foraging ecology of seabirds can help to understand their behavioural response to the variability of environmental conditions in the marine ecosystem [115]. Our interdisciplinary study combining telemetry and remote sensing (satellite imagery) provides a deeper insight into the foraging ecology of the Brunnich’s guillemot—one of the flagship High Arctic seabird species breeding in Svalbard—the polar region affected the most by climate change [40].

Range and duration of foraging trips of the studied chick-rearing guillemots are broadly consistent with results reported from other High and Low Arctic populations [7,58,60,81,116]. Mean trip duration reported in our study (approx. 12 h) was similar to the values from other High Arctic populations (approx. 12, 7 h) [58] (approx. 9 h) [58,116] and chick-rearing guillemots from Low Arctic (approx. 10 h) [60]. Also, mean distance from the colony to foraging areas in our study (approx. 42 km) was comparable to the potential foraging range reported for guillemot populations from other High Arctic colonies in Greenland (approx. 50 km [58], approx. 42 km [116], and Canada (approx. 50 km [58]). Similar ranges and flight characteristics in various guillemot populations may be attributed to the very high cost of foraging in guillemots [47]. Ranges of environmental conditions at foraging areas (especially SST and DEPTH) were also comparable to those reported for other populations [60,64]. SST range reported in our study (3–8°C) was similar to the range reported for guillemots from Low Arctic colony on St George Island, Alaska, i.e. (6.9–10.4°C) [60]. Birds also mostly used areas of similar depth near the colony, i.e. approx. 200–250 m as described from the Low Arctic colony in Iceland [64] and our results. Besides different environmental conditions between years, the studied guillemots did not show differences in any of the considered foraging flight characteristics, which indicates behavioural plasticity and adaptiveness of this species. No inter-annual variation in trip characteristics have also been reported for the common guillemot *Uria aalge* breeding on Southeast Farallon Island [61]. However, one should remember that we deployed GPS loggers, which may modify birds’ natural foraging behaviour. We also conducted this study on a small sample of individuals taken from the lowest cliff shelves which comprised only a small proportion of the large colony and was not representative of the population as a whole.

We found that the studied guillemots preferred to forage in cold water, mainly in the temperature range optimal for cold-water prey like polar cod [63,117,118] and cold-water amphipods and euphausiids, which can serve locally as an important component of the guillemots’ diet [53,119]. We have no data about the diet of the studied birds, but regurgitated food collected from black-legged kittiwakes *Rissa tridactyla* breeding in the same colony and sharing the same diet in other Svalbard colonies (e.g. [25,120,121] was characterized by high contribution of polar cod (84% samples), macroplanktonic euphausiids (44%) and hyperiids (24%) [25]). However, we did not investigate diet composition of instrumented individuals, which could have provided insight on food availability. To better understand the dynamics of the foraging niches of guillemots and other High Arctic seabirds representing different foraging guilds, further studies covering more years, all stages of reproduction, and several colonies from different regions of the Arctic are undoubtedly necessary.

In many studies, the importance of the marginal ice zone at sea for guillemots has been emphasized (e.g. [52,64,122]). The presence of sea ice promotes the availability of cold-water prey like polar cod [123]. The marginal ice zone is very productive due to deposition of the nutritional matter to the nearby waters (e.g. [25]). However, during the study period, no sea ice was observed within the range of the studied guillemot’s foraging trips [124] (electronic supplementary material, figure S2) [125]. Also, marine-terminating glaciers fronts may provide attractive foraging areas for many seabirds through meltwater discharge that generates upwellings of freshwater carrying their prey to the surface [25,126]. This does not apply to guillemots, which avoid feeding in turbid water [127], and in Hornsund, being a highly glaciated fjord [25,27], the distribution of guillemots based on at-sea surveys has been mainly restricted to the non-glaciated zones [128]. During our research, we did not detect any foraging locations of GPS-tracked individuals close to marine terminating glaciers (electronic supplementary material, figure S3).

The guillemots we examined in SW Spitsbergen mainly foraged at the shelf zone, but also at the shelf break zone. Shelf outside of the Hornsund fjord is dominated by the cold-water masses of Arctic origin (e.g. [65,67]) serving as an attractive habitat for cold-water organisms. Along the shelf break zone, a hydrological front (Arctic or Polar Front) is often situated, which separates the warmer Atlantic and Arctic water masses [129,130]. The range and distribution of both types of water masses vary considerably among the years [62,67]. Frontal zones by locally enhancing productivity and concentration of prey have been recognized as foraging hot spots for both planktivorous and piscivorous seabirds, including guillemots [21,22,131–138]. Some tracked individuals foraged in this zone regardless of year (electronic supplementary material, figure S4). The frontal zone may act as an alternative source of attractive food, especially in the condition of food shortage in warmer years or later in the season when local resources are reduced or depleted (temporal prey depletion halo effect) [47,59,60].

In contrast to our expectation, the foraging habitat niche of the studied guillemots in colder 2015 was significantly broader compared with warmer 2016. Broader habitat niche in 2015 was mainly driven by bigger range of SST and CHLA. It is very likely that the higher abundance and availability of prey in the colder 2015 year allowed guillemots to forage in a wider range of microhabitats. In contrast, in warmer 2016, studied birds presumably were forced to focus on restricted number of microhabitats still offering attractive prey like frontal zones (see electronic supplementary material, figure S1) or deep cold-water refugia (covered with warmer water masses in surface layers) like eddies or deep trough crossing the shelf (Hornsunddjupet) which are inhabited by cold-water prey including polar cod [53,64,67,136,137,139]. Smaller core and home ranges recorded in warmer 2016 with lack of inter-annual differences in foraging trips duration indicates that in that year birds allocated more time and energy to foraging without spatial expanding of foraging trips. This interpretation is supported by the higher diving activity of the GPS-tracked individuals in colder and prey abundant 2015, which suggests more frequent, shorter and shallower and therefore less energetically expensive dives to acquire food. In contrast, in warmer 2016, birds performed fewer dives per trip suggesting longer and presumably deeper thus more costly dives to capture prey persisting in cold-water refugia at greater depths. The more frequent diving activity observed during the more advanced chick-rearing period in both years of the study could be related to the higher energy requirements of the growing offspring [47]. Further studies including diving loggers could help to better understand observed foraging flexibility in response to divergent oceanographic conditions.

In both studied years, as the chick-rearing period progressed, distances between foraging locations and the colony increased. This observation suggests the presence of the temporal prey depletion halo effect [47,59,60]. This phenomenon is caused by depletion of available prey in closer foraging areas, forcing birds to search for food in further areas to maintain satisfactory energy provision to their growing offspring [54,140,141]. The facing temporal prey depletion halo by birds from Hornsund colony is supported by the fact that also with the progress of the breeding season, tracked guillemots exploited suboptimal foraging areas such as warmer waters with lower primary productivity. Furthermore, in the later stages of the breeding season, birds foraged at locations with greater depths. It may suggest that due to depletion of resources in shallower waters, they were searching for prey in harder-to-reach deep habitats such as eddies or troughs. It is also possible that guillemots hunted at greater depths to find larger prey for their growing offspring. All of the observed temporal differences in the environmental characteristics of the foraging locations, complemented by a higher distance between the foraging locations and the colony at more advanced stages of the chick-rearing period, confirm the presence of the temporal prey depletion halo effect. So far, such a phenomenon has been reported in guillemots breeding in Low Arctic (Coats Island, Canada), which hunted for pelagic prey items farther from the colony and collected less-energetic prey items later in the season [54].

## Data Availability

The dataset supporting this study is available online at OSF repository [143]. Supplementary material is available online [144].
